# The Impact of an Insecure Asylum Status on Mental Health of Adult Refugees in Germany

**DOI:** 10.32872/cpe.6587

**Published:** 2022-03-31

**Authors:** Victoria Sophie Boettcher, Frank Neuner

**Affiliations:** 1 Department of Clinical Psychology and Psychotherapy, Bielefeld University, Bielefeld, Germany; Philipps-University of Marburg, Marburg, Germany

**Keywords:** refugees, forcibly displaced people, mental health, post-traumatic stress disorder, insecure asylum status, post-migration living stressors

## Abstract

**Background:**

Forcibly displaced people have a higher chance of developing post-traumatic stress disorder (PTSD) compared to people who have not experienced displacement. In addition to potentially traumatic events due to war, persecution, and flight, post-migration living stressors are an important influencing factor. Among these, an insecure asylum status is one of the main stressors with which forcibly displaced people must cope. The aim of this study was to investigate the additive effect of an insecure asylum status on PTSD symptomatology in refugees, over and above the influence of other pre- and peri-migration factors, in particular potentially traumatic event types reported and duration of stay in Germany.

**Method:**

Two overlapping convenience samples of 177 and 65 adult refugees that were assessed at different timepoints were interviewed by means of face-to-face interviews. Interviews were conducted in either Arabic, Farsi, Kurmancî, English, or German with the assistance of interpreters where necessary. Besides residence status and potentially traumatic events experienced, mental distress was assessed via the Refugee Health Screener-15 (RHS-15; Study A) and the PTSD Checklist for DSM-5 (PCL-5; Study B).

**Results:**

In both samples, an insecure asylum status explained a significant additional amount of variance of PTSD symptomatology, on top of traumatic events experienced and time since arrival in Germany.

**Conclusion:**

Results suggest that refugees with an insecure asylum status are at higher risk for experiencing increased PTSD symptomatology. Policy changes of asylum procedure in receiving countries could have a positive impact on refugees’ mental health.

## Highlights

- Prevalence rates of mental disorders are high among forcibly displaced people.
- The impact of post-migration stressors on refugees’ mental health should not be disregarded.
- As one of the possible post-migration stressors, asylum status is substantially associated with mental health.
- Changes to reception policies may be taken into account.

## Background

At the end of 2019, 79.5 million people were forcibly displaced worldwide. Over the course of the previous decades this number has increased consistently (UNHCR, 2020). The high numbers pose serious challenges to the receiving countries, straining their capacity to provide housing, food, healthcare services, and education. As a result, during the last years, several potential receiving countries have adapted their reception policies regarding people seeking refuge (Fazel, Karunakara, & Newnham, 2014; Jakubowicz, 2016; Li, Liddell, & Nickerson, 2016). As a consequence, in 2019 less than 40% of asylum seekers were formally recognized as refugees (UNHCR, 2020).

Compared to non-refugees, those who have been forcibly displaced have a higher risk of mental disorders, most prominently post-traumatic stress disorder (PTSD) and depression (Bozorgmehr et al., 2016; Gäbel, Ruf, Schauer, Odenwald, & Neuner, 2006). Studies demonstrated that mental disorders among refugees come along with a high burden. Due to the symptoms, such as difficulty concentrating or sleeping problems, learning a new language, staying engaged in classes, or going to work on a regular basis can be much harder (Elbert, Wilker, Schauer, & Neuner, 2017).

Several studies have pointed out that post-migration stressors in the receiving countries have an impact on the onset and maintenance of psychological disorders (Chu, Keller, & Rasmussen, 2013; Li et al., 2016).

### Asylum Application Procedure in Germany

One of the most salient post-migration stressors is an insecure residence permit that may leave refugees living in uncertainty and with restricted rights for months and even years (Li et al., 2016). In Germany, there are several different types of residence status for refugees (Federal Office for Migration and Refugees, 2019). The *entitlement to asylum*, according to Article 16a para. 1 of the constitution (Grundgesetz), and the *refugee protection*, according to section 3 subs. of the Asylum Act (AsylG), involve similar implications for affected people’s lives. Both comprise a residence permit for three years. Access to the labor market is not restricted and the refugees are entitled to family reunification. Moreover, in case people meet preconditions like German language skills, a permanent settlement permit after three or five years is possible. According to section 4 subs. 1 of the Asylum Act (AsylG), *subsidiary protection* comprises a residence permit for one year, which can be repeatedly extended by two years. Similar to the two other forms of protection stated above, receiving a settlement permit is possible but only after five years. Access to the labor market is unrestricted as well. In contrast to the entitlement to asylum and the refugee protection, people with a subsidiary protection are not entitled to privileged family reunification. Individuals holding one of these three types of permits have a right to move to their own homes with some regional restrictions and a comparable health care protection as the general population in Germany.

Individuals who receive a *national ban on deportation* have a residence permit for at least one year, with possibility of extension. Again, receiving a settlement permit is possible after five years. In contrast to the other forms of protection stated above, there are restrictions regarding the access to the labor market. The same holds for asylum seekers with *pending applications*. When an asylum *application is turned down*, the person has to leave Germany in the near future.

### Consequences of an Insecure Residence Status for Mental Health in Refugees

During recent years, the potential influence of an insecure residence status on mental health in forcibly displaced people became increasingly apparent. Research findings tend to show that insecure status is correlated with mental health symptoms (Heeren et al., 2016; Müller, Zink, & Koch, 2018; Newnham, Pearman, Olinga-Shannon, & Nickerson, 2019). In a study by Momartin et al. (2006), residing under a temporary permit to stay was found to be the greatest predictor of PTSD symptomatology even when having accounted for trauma experiences in the analyses. However, some studies have provided mixed results (Schick et al., 2016; Winkler, Brandl, Bretz, Heinz, & Schouler-Ocak, 2019). Schick et al. (2016) found that, while PTSD symptomatology was correlated with a sum-score of other post-migration stressors, there was no isolated influence of visa status. Similarly, Winkler et al. (2019) did not find a significant association between visa status and PTSD symptoms. However, among participants who fulfilled PTSD criteria, symptom intensity was increased with an insecure asylum status.

As reported above, visa insecurity often comes with restrictions in daily life like limited access to health care services or limited rights (Müller et al., 2018). These factors seem to increase the risk of mental disorders (Chu et al., 2013) and complicate the process of integrating into a new society because opportunities to do so are limited (Müller et al., 2018). These findings are supported by previous research that found that mental health improved following the granting of a residence permit (Lamkaddem, Essink-Bot, Deville, Gerritsen, & Stronks, 2015).

Next to visa status, the duration of stay in the host country (Nickerson et al., 2019) that is highly associated with the duration of the asylum procedure (Laban, Gernaat, Komproe, van der Tweel, & De Jong, 2005) may have an influence on mental health of refugees and people seeking asylum. In a study with refugees in Australia, duration of stay was correlated with suicidal intent (Nickerson et al., 2019). An association of duration of asylum procedure and anxiety disorders was found by Laban et al. (2005). However, findings in the literature have been unable to confirm a consistent association, since other studies have found no effect (Heeren et al., 2016; Winkler et al., 2019). It may be conceivable that duration of stay assumes central importance only when it exceeds a threshold value. Differentiating between different aspects of post-migration stressors, Laban, Komproe, Gernaat, and de Jong (2008) concluded that asylum seekers who had been in the Netherlands for more than two years had several post-migration stressors to cope with, which might explain the association they made in earlier research (Laban et al., 2005) regarding the length of stay in the receiving country with mental health.

Although the nature of the association between asylum seekers’ mental health and their length of stay in their host context remains uncertain, it is clear that forcibly displaced people often encounter significant stressors and have limited access to coping resources because of their pre- and peri-migration experiences, new living situation, and post-migration stressors. Research has shown that the stressors experienced by people seeking asylum and recognized refugees can be divided in two categories (Womersley, Kloetzer, & Goguikian Ratcliff, 2017). The first category is associated with difficulties with housing and labor, which are reported by both groups. The second category is experienced more acutely by asylum seekers, who have reported uncertainty, lack of control, and insecurity. Asylum seekers live under constant threat of being expelled from their relatively safe living environment (Müller et al., 2018). Uncertainty is one of the factors increasing the probability of continuing mental disorders (Bogic et al., 2012; Ryan, Benson, & Dooley, 2008) and personal control is lost (Ryan, Benson, & Dooley, 2008). Therefore, no complete security can be felt, which seems to be closely related with the development and maintenance of mental distress/PTSD.

### Aim of the Study

This study seeks to investigate the effects of asylum status on PTSD symptomatology over and above the influence of potentially traumatic event types reported and length of stay in country of arrival in refugees living in Germany.

## Method

In this paper, two studies and the respective results are presented. *Study A* and *Study B*, including procedures and measures used, will be presented successively. The samples are overlapping and Sample B is a detailed and more comprehensive re-assessment of a subset of Sample A. All participants of *Study A* who consented to a second interview were tried to be reached via telephone, email, or in person. Participants in Sample B participated in additional clinical face-to-face interviews by an expert interviewer that allowed to apply more detailed clinical scales some months after the first interview.

### Sample

*Study A.* Between February and August 2018 face-to-face interviews were conducted with 198 refugees (23.2% female, *n* = 46). The unselected convenience sample ranged from 18 to 75 years of age (*M* = 33.03, *SD* = 11.02). Refugees were eligible to participate if they were at or above the age of majority, were living in North Rhine-Westphalia, had sufficient language skills to be able to conduct the interview in Arabic, Farsi, Kurmancî (as these three languages were the most common ones on site at the time of the study), English or German, and their time since arrival in Germany did not exceed six years.

*Study B.* Between August 2018 and March 2019, refugees form *Study A* were recontacted and approached for interviews. Out of these, 65 refugees (20.0% female, *n* = 13) participated, the remaining could not be contacted or were not available for a re-interview. The participation rate of 32.8% may be explained by the fact that a substantial proportion of the participants of *Study A* had no secure residence status and had possibly been forced to leave Germany in the meantime. In general, most participants could be contacted successfully via telephone, email, or in person were consenting to take part in *Study B*. Participants ranged from 19 to 75 years of age (*M* = 34.50, *SD* = 12.13).

### Procedures

Data collection was conducted within the framework of the research consortium “FlüGe–Opportunities and challenges that global refugee migration presents for health care in Germany” and was part of a larger study. The program was funded by the Ministry of Culture and Science of the State of North Rhine-Westphalia, Germany. Thirteen paraprofessional interviewers (12 male, 1 female) were trained as both interviewers and interpreters (9 Arabic native speakers, 3 Farsi native speakers, 4 Kurmancî native speakers). Data was collected in a region in the north-east of North Rhine-Westphalia, Germany. Interviews took place in shared accommodation facilities, private apartments, and on the Bielefeld University campus. Potential literacy problems were avoided by reading out all questions to participants. The respondents were free not to answer single questions without giving reasons. The Ethical Review Board of Bielefeld University granted approval for the study. To ensure the voluntariness of participation in an interview that may provoke distress in some individuals no compensation was provided for participation.

*Study A.* All material (informed consent forms, information letters, questionnaire) was translated by a professional translation agency and native speakers. Blind back-translations ensured correct translation. Participants were identified through contact with social workers who have been working in the region and made contact with the shared accommodations. During informational events, initial interview appointments were arranged. Further appointments were agreed on by asking people present in the accommodations and via snowball sampling. Field teams consisted of two supervising researchers and the necessary interviewers. The face-to-face interviews lasted on average 90 minutes (*SD* = 31.9).

*Study B.* Informed consent forms and information letters were again translated and blind back translated. In the event that participants had previously provided their written consent and contact information, they were contacted via telephone, email, or home visits. Face-to-face interviews were conducted by German speaking researchers with the assistance of interpreters where necessary. Interviews lasted 116 minutes on average (*SD* = 48.2). Participants were interviewed an average of six months after they had been interviewed for *Study A*.

### Measures

*Study A.* Information regarding age, gender, citizenship, education, marital status, length of time since arrival in Germany, and potentially traumatic event types was collected (see Appendix A in the Supplementary Materials). In addition, mental distress and residence status were assessed (see detailed description below).

*Study B.* In addition to the questions assessed in the first interview, participants were asked to answer further questions regarding potentially traumatic event types and PTSD symptomatology. Residence status was assessed again (see detailed description below).

#### Mental Distress

*Study A.* The 15 item Refugee Health Screener-15 (RHS-15; Hollifield et al., 2013) assesses mental distress in refugees. The first 13 questions assess the presence of different symptoms of depression, anxiety, and PTSD during the last month. Question 14 measures the general coping capacities. Answers are given on a 5-point Likert scale (not at all – extremely). Question 15 assesses how much suffering the participant experienced last week. Responses to this item were reported on a scale of 0–10 on a “distress thermometer”. Effectiveness, validity, and reliability of the screening instrument have been demonstrated in various studies (Hollifield et al., 2016; Hollifield et al., 2013; Kaltenbach, Härdtner, Hermenau, Schauer, & Elbert, 2017). In *Study A* a Cronbach's α of .87 was found. The cutoff value recommended by Hollifield et al. (2013) is a sum-score of ≥ 12 regarding questions 1–14 and/or a score of ≥ 5 regarding the distress thermometer. The former cutoff is used in this study. Regarding this cutoff, a sensitivity of = .81 and specificity of = .87 for PTSD was reported (Hollifield et al., 2013).

*Study B.* The German version of the Posttraumatic Stress Disorder Checklist for DSM-5 (PCL-5; Krüger-Gottschalk et al., 2017) was used to assess PTSD symptomatology within the past month. The PCL-5 consists of 20 questions. Answers were rated 0 (not at all) – 4 (extremely), which results in the highest possible score of 80. In the current sample, Cronbach’s α was .86. Good psychometric properties have been demonstrated in previous studies (Krüger-Gottschalk et al., 2017; Wortmann et al., 2016). Ibrahim, Ertl, Catani, Ismail, and Neuner (2018) used the translated checklist in displaced Arab and Kurdish populations and came up with a cut-off score of 23 to be the best balance between specificity and sensitivity in these populations.

#### Residence Status

The answers regarding the question assessing residence status were grouped in six categories (recognized as refugee, entitled to asylum, subsidiary protection, asylum applicant with pending procedure, temporary suspension of deportation, demand to leave Germany). The first three of the categories were classified as “secure residence status”. The latter three were classified as “insecure residence status”.

### Data Analysis

Statistical analyses were performed with IBM SPSS Statistics Version 27 for macOS. Due to ≥ 10% missing data in the RHS-15, 21 participants were excluded from *Study A*. Regarding cases with < 10% missing values on the RHS-15, values were set equal to 0. Multiple linear regression analyses with two levels were carried out for both samples. In *Study A*, the RHS sum-score to assess mental distress and in *Study B*, the PCL-5 sum-score to assess PTSD symptomatology were used as dependent variables. Both analyses accounted for age, gender (females coded as 0, males coded as 1), number of traumatic event types reported, and time (in month) since arrival in Germany. The variables accounted for were entered in step one. The dummy coded residence status (secure residence status coded as 0, insecure residence status coded as 1) was added in the second step. For the analyses the alpha level was set at 0.05.

## Results

*Study A.* The 177 participants (20.3%; *n* = 36 female) were, on average, 33 years old (*SD* = 11.21). With 42.4% (*n* = 75) the largest proportion of participants had a Syrian citizenship, followed by 26.6% (*n* = 47) with an Iraqi citizenship, and 9.0% (*n* = 16) with an Afghan citizenship. The average time since arrival in Germany was 28.5 months (*SD* = 9.96). An insecure residence status was reported by 30.5% (*n* = 61) of participants. RHS mean sum-score (Items 1–14 of the RHS-15) was 15.61 (*SD* = 10.92). A score above the cutoff (score ≥ 12) was reached by 54.8% (*n* = 97) of the participants.

*Study B.* The 65 participants (20.0%, *n* = 13 female) were, on average, 35 years old (*SD* = 12.13). The majority stated holding a Syrian citizenship (58.5%, *n* = 38), followed by 23.1% (*n* = 15) with an Iraqi citizenship. Average time since arrival in Germany was almost three years (*M* = 34.66 month; *SD* = 10.68). An insecure residence status was indicated by 15 participants (16.5%; see Appendix A in the Supplementary Materials for all descriptive data). The mean score on the PCL-5 was 19.68 (*SD* = 14.58). Using a suggested cut-off score of 23 for Arabic and Kurdish displaced populations (Ibrahim et al., 2018), 25 participants (38.5%) met DSM-5 criteria for probable PTSD diagnosis.

### Mental Distress

*Study A*. Participants who indicated having an insecure residence status had a higher RHS-15 sum-score (*M* = 20.52; *SD* = 11.53) compared to participants holding a secure residence status, *M* = 13.00; *SD* = 9.76; *t*(171) = −4.54, *p* < .001.

*Study B*. Participants holding an insecure residence status reported an average score of 30.67 (*SD* = 15.98) on the PCL-5, whereas participants with a secure residence status scored 16.38 (*SD* = 12.52) on average, *t*(63) = −3.63, *p* = .001 (see Appendix A in the Supplementary Materials for all descriptive data).

### Residence Status

Of the 177 participants in *Study A*, four participants did not indicate their residence status, 61 indicated having a relatively secure residence status (34.5%). In *Study B*, 23.0% of participants reported an insecure residence status (see Table 1 for a detailed overview).

**Table 1:** Descriptive Statistics of Participants’ Residence Status Type

Residence status	Study A (*N* = 177)		Study B (*N* = 65)	
	*n*	%	*n*	%
Secure residence status				
Recognized as refugee	42	23.7	6	9.2
Entitled to asylum	35	19.8	24	36.9
Subsidiary protection	35	19.8	20	30.8
Insecure residence status; *n* (%)				
Asylum applicant with pending procedure	38	21.5	6	9.2
Temporary suspension of deportation	19	10.8	8	12.3
Demand to leave Germany	4	2.3	1	1.5
**Missing**	4	2.3	0	0.0

*Note.* % figures rounded to one decimal place.

### The Impact of Residence Status on Mental Health–Multiple Regression Analyses

*Study A.* Variables added in the first step (age, gender, number of event types reported, time (in month) since arrival in Germany) resulted in an *R*^2^ of .12 (*p* < .001). The variables accounted for a significant amount of variance of mental distress variability. Adding residence status in the second step explained an additional 7.7% of variance (∆*R*^2^ = .08, *p* < .001). In the first step, age, gender, and number of traumatic event types experienced were significantly associated with the RHS sum-score. In the second step, gender, number of reported event types, and residence status were significantly associated with the RHS sum-score. An insecure residence status was associated with a higher RHS sum-score. Overall, a significant regression equation was found, *F*(5, 161) = 7.82, *p* < .001. The final model accounted for 19.5% of the total variance in mental distress captured by the RHS-15 (see Table 2 for exact values).

**Table 2:** Hierarchical Regression Analysis of PTSD Symptoms

Variable	Study A (RHS-15 sum-score as dependent variable)^a^		Study B (PCL-5 sum-score as dependent variable)^b^	
	*B* [95% CI]	*p*	*B* [95% CI]	*p*
Step 1				
Age	0.15 [0.00, 0.30]	.046*	0.12 [-0.14, 0.39]	.351
Gender	-5.47 [-9.61, -1.33]	.010*	-15.29 [-23.70, -6.88]	.001*
Number traumatic event types reported	0.92 [0.45, 1.30]	< .001*	1.02 [0.37, 1.68]	.003*
Time since arrival in Germany^c^	-.04 [-0.22, 0.14]	.657	0.49 [0.17, 0.82]	.004*
Step 2				
Age	0.14 [-0.01, 0.28]	.059	0.16 [-0.08, 0.40]	.187
Gender	-5.35 [-9.32, -1.38]	.009*	-16.33 [-24.09, -8.56]	< .001*
Number traumatic event types reported	0.67 [0.20, 1.14]	.005*	0.71 [0.09, 1.34]	.026*
Time since arrival in Germany^a^	-0.04 [-0.21, 0.14]	.640	0.45 [0.14, 0.75]	.004*
Insecure residence status	6.65 [3.30, 10.00]	< .001*	12.38 [5.13, 19.63]	.001*

^a^*R*^2^ = .12 for Step 1 (*p* < .001); ∆R^2^ = .08 for Step 2 (*p* < .001). Listwise deletion. *N* = 167. ^b^*R*^2^ = .34 for Step 1 (*p* < .001); ∆*R*^2^ = .11 for Step 2 (*p* = .001). Listwise deletion. *N* = 64. ^c^in month.

*p ≤ .05.

*Study B.* Variables added in the first step accounted for 34.4% of variance of PTSD symptomatology (*R*^2^ = .34, *p* < .001). By adding residence status in the second step additional 11.0% of variance of the PCL-5 sum-score was explained (∆*R*^2^ = .11, *p* = .001). Apart from age, all variables were significantly associated with the PTSD symptom variability in both steps of the regression analysis. A significant regression equation was found, *F*(5, 58) = 9.64, *p* < .001. The final model accounted for approximately 45.4% of the total variance of PTSD symptomatology (see Table 2 for exact values).

## Discussion

### Impact of Residence Status on Refugees’ Mental Health

Our study of forcibly displaced people found that people with an insecure asylum status are at higher risk for an increased PTSD symptomatology. These findings are in line with earlier research (Heeren et al., 2016; Müller et al., 2018; Newnham et al., 2019). Potential explanations for our results must include a consideration of the kind and amount of post-migration stressors experienced by the refugees. As described by Womersley et al. (2017), people with an insecure asylum status often have a larger number of stressors than those with a more secure asylum status.

Female gender was accompanied with increased symptomatology scores (5.35 on the RHS-15 and 16.33 on the PCL-5). This finding is in line with earlier research reporting female gender as a predictor of PTSD symptomatology (Mahmood, Ibrahim, Goessmann, Ismail, & Neuner, 2019; Nickerson et al., 2019). As females did not report a significantly higher number of potentially traumatic event types, we assume that it may be the type of trauma rather than simply the number that is associated with an increased mental stress/PTSD symptomatology score. Moreover, other factors like perceived social support may play a role here. The fact that participants in our studies holding an insecure asylum status reported having experienced a higher number of traumatic event types, on average, is in line with earlier studies as well (e.g., Nickerson et al., 2019). For every additional event type reported, the RHS-15 and PCL-5 sum-scores increased by 0.67 in *Study A* and 0.71 points in *Study B*. Participants with an insecure asylum status reached an RHS-15 sum-score 6.65 points higher than participants with a secure asylum status. The PCL-15 sum-score was 12.38 points higher for participants with an insecure status. Duration of stay was only significant in *Study B*. A possible explanation is that participants who took part in *Study B* had spent on average six months longer in Germany and thus had a longer exposure to post-migration stressors. Furthermore, the different finding may be explained by the different questionnaires used in the studies.

### Strength and Limitations

An advantage of having two different studies is the potential for participants to develop an increased level of trust with research staff by taking part in *Study B*. This aspect was emphasized by statements of some of the participants describing a joy to meet again. By collecting all information through face-to-face interviews instead of (online) questionnaires, possible difficulties in comprehension could be resolved.

Our study is based on convenience samples that, although unselected, are far from representative of refugee populations in Germany, which limits the generalizability of the findings. However, the fact that the same associations were found consistently across two measurements with different instruments supports validity of findings.

Data collection was cross-sectional in both studies. To be able to increase explanatory power and investigate causation longitudinal studies are needed. Even though the RHS-15 is a well-known screening tool with good reliability and validity scores, it is a screening tool with 15 items and does not allow a more detailed insight in a person’s mental health status or the diagnosis of potential mental health disorders.

### Recommendations

In line with previous research (Chu et al., 2013), we found that asylum status as a post-migration factor explains a significant amount of variance in PTSD symptomatology. It seems evident that post-migration conditions can interfere with recovery from traumatic experiences (Heeren et al., 2014). People are best positioned to thrive when they experience a safe environment to be able to profit from available resources (Ryan et al., 2008). Further research on post-migration stressors could provide more insight in the potential influence of these stressors on PTSD symptomatology. Moreover, including additional measures apart from RHS-15 and PCL-5 to investigate mental health status could offer an even more comprehensive insight in refugees’ mental health. Lastly, investigating the possible confounding associations of citizenship with asylum status and mental distress may be insightful. However, to make reliable statements and draw conclusions, a larger sample size with a more balanced distribution of citizenships as well as residence status types will be needed.

Refugees need the opportunity to participate in everyday life. With a working permit for integration, learning a new language, making socially supportive contacts, the PTSD rate decreases (Hocking, Kennedy, & Sundram, 2015). Policy changes regarding the asylum procedure in receiving countries could therefore have a positive impact on refugees’ mental health (Porter & Haslam, 2005). As long as asylum procedures cannot be substantially shortened, freedom of movement and access to the labor market should be provisionally granted. These changes may relieve at least some post-migration stressors.

Furthermore, the possible influence of an insecure asylum status on psychotherapy needs to be considered (Chu et al., 2013). The additional stress might impede the therapeutic process. The increased risk of symptoms becoming chronic and the accompanying higher costs for the health care system could be bypassed by granting unconditional access to the health care system regardless of asylum procedure. Knowing about the negative aspects of post-migration living stressors it might be also interesting to consider the opposite side, namely whether easing post-migration living conditions can promote recovery and growth.

### Conclusions

It is not only the potentially traumatic events experienced before or during flight that have an impact on refugees’ mental health. In fact, conditions in the receiving countries contribute to psychological well-being. To be able to expect successful integration, opportunities for inclusion in everyday life need to be offered. Changes in residence status policies may be one step in the right direction. Apart from the people going through the established asylum procedure, forcibly displaced people who immigrated illegally should be kept in mind as well. Healthcare services should not be hold back for people suffering from physical or mental illness regardless of asylum status. By providing refugees opportunities to be independent and active members of their communities, both they and society at large stand to benefit as refugees have a clearer path to realizing their potential.

## Supplementary Materials

In the Supplementary Materials, a table with descriptive statistics of all relevant variables is displayed (for access see Index of Supplementary Materials below).

10.23668/psycharchives.5412 Supplement 1 Supplementary materials to "The impact of an insecure asylum status on mental health of adult refugees in Germany"

BoettcherV. S.
NeunerF.
 (2022). Supplementary materials to "The impact of an insecure asylum status on mental health of adult refugees in Germany"
[Descriptive statistics]. PsychOpen. 10.23668/psycharchives.5412
PMC966734536397747
