# Supplementary material for: The Impact of an Insecure Asylum Status on Mental Health of Adult Refugees in Germany
Source: Clin Psychol Eur. 2022 Mar 31;4(1):e6587. doi: 10.32872/cpe.6587 (PMC9667345; doi:10.32872/cpe.6587)
Supplement: Supplement 1 [file cpe-04-6587-s01.pdf]

Supplementary material to the article “The Impact of an Insecure Asylum Status on Mental Health of Adult Refugees in Germany”

DOI link: https://doi.org/10.32872/cpe.6587

Authors: Victoria Sophie Boettcher, Frank Neuner

Appendix A

Table A. Descriptive Statistics of all Relevant Variables.

	Study A			Study B		
	Total sample (*N* = 177)	Subsample with a secure residence status (*n* = 112)[a]	Subsample with an insecure residence status (*n* = 61)[b]	Total sample (*N* = 65)	Subsample with a secure residence status (*n* = 50)[a]	Subsample with an insecure residence status (*n* =15)[b]
Age (in years)						
M (SD)	33.10 (11.18)	32.81 (11.10)	33.30 (11.36)	34.50 (12.13)	35.36 (12.54)	31.56 (11.00)
Range	18–75	18–75	19–63	19–75	19–75	20–61
Gender (female); *n* (%)	36 (20.3)	24 (21.4)	12 (19.7)	13 (20)	12 (24.0)	1 (6.7)
Formal education; *n* (%)						
Dropped out of school without certificate	30 (16.9)	18 (16.1)	11 (18.0)	6 (9.2)	3 (6.0)	3 (20.0)
Primary school graduation	34 (19.2)	21 (18.8)	13 (21.3)	11 (16.9)	8 (16.0)	3 (20.0)
Secondary school certificate	25 (14.1)	14 (12.5)	9 (14.8)	10 (15.4)	7 (14.0)	3 (20.0)
High school graduation	74 (41.8)	53 (47.3)	20 (32.8)	34 (52.3)	30 (60.0)	4 (26.7)
Higher education[c]; *n* (%)	37 (20.9)	27 (24.1)	10 (16.4)	18 (27.7)	15 (30.0)	3 (20.0)
Marital status; *n* (%)						

In a stable partnership (married or unmarried)	107 (60.5)	72 (64.3)	31 (50.9)	37 (57.0)	30 (60.0)	7 (46.7)
Citizenship (multiple answers possible); *n* (%)						
Syria	75 (42.4)	73 (65.2)	2 (3.3)	38 (58.5)	37 (74.0)	1 (6.7)
Iraq	47 (26.6)	19 (17.0)	26 (42.6)	15 (23.1)	10 (20.0)	5 (33.3)
Afghanistan	16 (9.0)	4 (3.6)	12 (19.7)	6 (9.2)	0 (0.0)	6 (40.0)
Other	38 (21.5)	15 (13.4)	21 (34.4)	6 (9.2)	3 (6.0)	3 (20.0)
Time since arrival in Germany in months						
M (SD)	28.46 (9.96)	28.32 (10.25)	28.83 (8.93)	34.66 (10.68)	33.32 (11.24)	39.13 (7.14)
Range	1–63	3–63	4–49	8–57	8–56	24–57
Potentially traumatic event types						
M (*SD*)	6.97 (3.56)	6.27 (3.31)	8.28 (3.69)	11.05 (5.01)	10.10 (4.87)	14.13 (4.31)
Range	0–19	0–19	2–18	1–21	1–20	7–21
Refugee Health Screener-15 (Question 1-14); *M* (*SD*)	15.61 (10.92)	13.00 (9.76)	20.52 (11.53)	n.a.	n.a.	n.a.
PCL-5 sum-score; *M* (*SD*)	n.a.	n.a.	n.a.	19.68 (14.58)	16.38 (12.52)	30.67 (15.98)

Note. % figures rounded to one decimal place.

[a] recognized as refugee, entitled to asylum, subsidiary protection

[b] asylum applicant with pending procedure, temporary suspension of deportation, demand to leave Germany

[c] Diploma, bachelor’s degree, master’s degree, PhD, postdoctoral qualification
